# Comparative development and ocular histology between epigean and subterranean salamanders (*Eurycea*) from central Texas

**DOI:** 10.7717/peerj.11840

**Published:** 2021-07-28

**Authors:** Ruben U. Tovar, Valentin Cantu, Brian Fremaux, Pedro Gonzalez Jr, Amanda Spikes, Dana M. García

**Affiliations:** 1 Department of Biology, Texas State University, San Marcos, TX, United States of America; 2 Department of Integrative Biology, University of Texas at Austin, Austin, TX, United States of America; 3 San Marcos Aquatic Resources Center, U.S. Fish and Wildlife Service, San Marcos, TX, United States of America; 4 Uvalde National Fish Hatchery, U.S. Fish and Wildlife Service, Uvalde, TX, United States of America

**Keywords:** Ocular development, Divergent evolution, Evolution and development, Subterranean salamander

## Abstract

The salamander clade *Eurycea* from the karst regions of central Texas provides an ideal platform for comparing divergent nervous and sensory systems since some species exhibit extreme phenotypes thought to be associated with inhabiting a subterranean environment, including highly reduced eyes, while others retain an ancestral ocular phenotype appropriate for life above ground. We describe ocular morphology, comparing three salamander species representing two phenotypes—the surface-dwelling Barton Springs salamander (*E. sosorum*) and San Marcos salamander (*E. nana*) and the obligate subterranean Texas blind salamander (*E. rathbuni*) - in terms of structure and size of their eyes. Eyes were examined using confocal microscopy and measurements were made using ImageJ. Statistical analysis of data was carried out using R. We also provide a developmental series and track eye development and immunolocalization of Pax6 in *E. sosorum* and *E. rathbuni*. Adult histology of the surface-dwelling San Marcos salamander (*E. nana*) shows similarities to *E. sosorum*. The eyes of adults of the epigean species *E. nana* and *E. sosorum* appear fully developed with all the histological features of a fully functional eye. In contrast, the eyes of *E. rathbuni* adults have fewer layers, lack lenses and other features associated with vision as has been reported previously. However, in early developmental stages eye morphology did not differ significantly between *E. rathbuni* and *E. sosorum*. Parallel development is observed between the two phenotypes in terms of morphology; however, Pax6 labeling seems to decrease in the latter stages of development in E.*rathbuni*. We test for immunolabeling of the visual pigment proteins opsin and rhodopsin and observe immunolocalization around photoreceptor disks in *E. nana* and *E. sosorum*, but not in the subterranean *E. rathbuni*. Our results from examining developing salamanders suggest a combination of underdevelopment and degeneration contribute to the reduced eyes of adult *E. rathbuni*.

## Introduction

Subterranean environments exert strong selective pressures on organisms that occupy them. Additionally, relaxed selection can be equally pervasive when an organism invades a novel subterranean niche. Both scenarios seem to apply to vertebrates that occupy subterranean environments (Rétaux & Casane, 2013), resulting in their convergent evolution. Arguably, a quintessential phenotype of subterranean organisms is the reduction of the eye, which may reflect a combination of relaxed selection, directional selection, and genetic drift (Rétaux & Casane, 2013). Eye reduction can be observed in organisms that occupy both terrestrial and aquatic subterranean environments. Terrestrial organisms that occupy dim light environments such as caves (troglobites) and fossorial niches share in a range of eye reduction, as is the case with cave beetles and some fossorial mammals, such as moles (Rétaux & Casane, 2013).

Obligate aquatic subterranean fauna are referred to as stygobites (Goricki, Niemiller & Fenolio, 2012). Similar to troglobites, stygobitic morphology includes drastically reduced eyes and pale skin; however, aquatic environments differ from terrestrial environments (e.g., conductivity) and could impose different selective pressures (Rohner et al., 2013). Stygobitic vertebrates broadly fall into two major lineages, teleost and Caudates. Within Caudata, the genus *Eurcyea* and *Proteus* show several occurrences of subterranean invasions. Two phenotypes have been recognized within the central European *P. anguinus* populations (*P. anguinus anguinus* and *P. anguinus parkelj*), showing differences in pigmentation and eye development with the former lacking pigmentation and eyes, and the latter being fully pigmented and exhibiting relatively more eye structure (Durand, 1976). Stygobitic morphology is exemplified in the genus *Eurycea* by the Texas blind salamander (*E. rathbuni*) with its reduced pigment and eye structure (Mitchell & Reddell, 1965). In contrast, the San Marcos salamander (*E. nana*) and Barton Springs salamander (*E. sosorum*) are surface species and have pigmented skin and seemingly well-developed eyes.

Ocular histology has been investigated in several families of salamanders (Fite, 1976; Linke, Roth & Rottluff, 1876; Roth, 1987), and differing degrees of ocular regression are documented in the subterranean species of the genera *Eurycea* (Eigenmann, 1900; Emerson, 1905), *Eurycea spelaea* (formerly in the genus *Typhlotriton*; Walls, 1942), and *Proteus* (Möller, 1951). Among the cave dwelling species, the eye reduction observed in *E. rathbuni* is relatively extreme: the eye, completely surrounded by melanized tissue, lacks the distinct layers observed in organisms with vision. Eye reduction in *Proteus anguinus anguinus*, while not fully surrounded by pigment, nevertheless has a reduced photoreceptor population. In contrast, *E. spelaea* individuals experience partial eye reduction during and post metamorphosis (Walls, 1942). Although ocular histology has been examined in *E. rathbuni* (Eigenmann, 1900), no direct comparisons to surface relatives have been made, nor have the developmental processes leading to morphological divergence been examined. Such a comparison is of interest because it could shed light on the interaction between evolutionary and developmental processes that lead to the divergence of closely related species. Significantly, there have been a number of subterranean invasions by the central Texas *Eurycea*, and phylogenetic analyses show strong support for a close relationship between the species with divergent ocular phenotypes (Bendik et al., 2013; Chippindale et al., 2000; Wiens, Chippindale & Hillis, 2003; Devitt et al., 2019).

The *Astyanax mexicanus* system exemplifies closely related populations, an epigean population and a styobitic (cavefish) population, with extremely divergent ocular phenotypes. Work by Jeffery et al. (2009) has shown that the loss of the eye in the cavefish can be partially rescued by transplanting the lens of an above-ground animal into a developing cavefish eye (Krishnan & Rohner, 2017). Furthermore, Jeffery (2005) has shown that loss of the eye in the cavefish is accompanied by upregulation of *sonic hedgehog* (*Shh*) and down-regulation of *paired homeobox protein-6* (*pax6*) preceding apoptosis of the lens tissue. In many species, Pax6 functions as a transcription factor and plays a role in the development of the anterior/posterior axis, the nervous system, and critically in eye development (Wawersik & Maas, 2000). Pax6′s diminished expression leads to eye reduction in the Somalian teleost cavefish *Phreatichthys andruzzii*. Unlike *A. mexicanus*, *P. andruzzii* experiences retinal apoptosis driven by waves of apoptotic events during late retinal development (Stemmer et al., 2015), revealing yet another process by which eye reduction may be accomplished.

Herein we describe for the first time the ocular histology of two surface species of central Texas *Eurycea*, namely *E. nana* and *E sosorum*.  Additionally, we compare the ocular histology of the subterranean *E. rathbuni* to that of the surface species, augmenting the descriptive study provided by Eigenmann (1900). We use immunohistochemistry to test for presence of the visual pigments opsin and rhodopsin in the eyes of adult *E. rathbuni*, *E. sosorum* and *E. nana*. We compare the size of eyes in developing and adult salamanders and discuss whether the reduced eyes observed in *E. rathbuni* represent underdeveloped eyes or regressed eyes. Finally, we present the first developmental series for *E. sosorum* and *E. rathbuni*, and use immunohistochemistry to compare expression of Pax6, which has been shown to be divergent in surface and cave populations of Mexican tetra (*A. mexicanus*; Jeffery, 2009) and Somalian cavefish (*P. andruzzii*; Stemmer et al., 2015). Given the importance of Pax6 during eye development in amphibians (Chow et al., 1999), we hypothesize that downregulation of Pax6 may be important in the development of stygomorphy in salamanders. Parallels in Pax6 expression between salamanders and fishes are discussed.

## Materials & Methods

### Specimens

The San Marcos Aquatic Resource Center (SMARC), Texas, United States Fish and Wildlife Service (USFWS) donated freshly dead adult specimens of Texas blind salamander (*Eurycea rathbuni*; *n* =  3), San Marcos salamander (*E. nana*; *n* =  3), and Barton Springs salamander (*E. sosorum*; *n* =  3) to Texas State University in San Marcos, Texas. The specimens’ heads were removed and transported to Texas State University for further processing under scientific permit number SPR-0390-045. General measurements along with tissue samples were taken from the remaining body which was then preserved in 95% ethanol and catalogued at SMARC. Embryos of *E. rathbuni* and *E. sosorum* were obtained from captive populations at SMARC. Staging was determined by morphology (Duellman & Trueb, 1986), and embryos were imaged using a stereomicroscope equipped with a camera. All animal manipulations were approved by Texas State University Institutional Animal Care and Use Committee (IACUC protocol approval number 0222_0530_12).

### Fixation and imaging

Techniques for fixation of heads and embryos followed Neve et al. (2012) as described below. Tissues were placed in 4% buffered paraformaldehyde for 24 h and washed three times for 10 minutes with phosphate buffered saline (PBS). Following fixation, tissues were placed in a 30% sucrose solution prepared in PBS for cryoprotection and stored at 4 °C for at least 24 h. Adult tissue sections were cut at 20 µm, mounted on a slide using 90% glycerol, and stored at −20 °C (Saul, Koke & García, 2010). At the conclusion of the study, sections were deposited at The University of Texas at Austin’s Biodiversity Center. Images were acquired using an Olympus FV1000 equipped with differential interference contrast optics and a 10×  objective.

### Retinal and ocular measurements

Images of ocular sections were opened in ImageJ software (Schneider, Rasband & Eliceiri, 2012), and the measurement tool was calibrated to each image. One image from each individual representing the three species (*E. rathbuni, n* =  3; *E. nana, n* =  3; and *E. sosorum, n* = 3) was selected for measurement. For the epigean species (*E. nana* and *E. sosorum*), the selection of the image was based on the presence of a lens in the section and six clearly distinguishable retinal layers: photoreceptor/retinal pigment epithelial layer, outer nuclear layer (ONL), outer plexiform layer (OPL), inner nuclear layer (INL), inner plexiform layer (IPL), and retinal ganglion cell layer (RGL). Measurements of retinal width were obtained from a region where the OPL appeared undistorted, signifying that the section in that region was not oblique. Three measurements were taken per individual with the transect being orthogonal to the OPL. Three measurements for each retinal layer were also obtained from each individual in the region of the transect. The means of the triplicate measurements were used to provide an estimate of thicknesses for that individual, and the three individuals provided an estimate of population means for their respective species (*n* =  3).

Thirty-four adult and three early developmental stage specimens for each species (*E. sosorum* and *E. rathbuni*) were obtained from SMARC and imaged using a Nikon D7000. Eye and head length measurements were obtained using ImageJ (File S1). Both eye and head measurements of each species were tested for normality. An analysis of variance (ANOVA) was conducted using eye measurements taken from adults and earlier developmental stages (standardized by head length) for each of the two species.

### Immunohistochemistry and imaging

Immunohistochemistry using transverse sections of embryo eyes was accomplished by blocking with 3% bovine serum albumin (Sigma Aldrich, A7030-10G) dissolved in PBS for two hours, then washing three times for ten minutes with PBS with 0.05% Tween. Sections were incubated with biotinylated anti-rat, mouse and chicken Pax6 antibody (R&D Systems Inc., #BAM1260) at a final concentration of 20 µg/mL for two hours at room temperature and with Cy5-conjugated streptavidin (Invitrogen, #43-8316, diluted 1:50) for two hours at room temperature. Two fifteen-minute washes were implemented between each incubation period using PBS. Negative controls for antibody labeling underwent identical processing except that no primary antibody was added. Coverslips were mounted in 90% glycerol, and slides were stored at 4 °C until imaged. Images were obtained using an Olympus FV-1000 scanning confocal microscope. Confocal settings were initially optimized on an *E. sosorum* sample, and settings remained constant while acquiring each successive image. Negative controls for Pax6 staining can be found in File S2.

Immunohistochemistry used to detect visual pigments in transverse sections of adult *E. rathbuni*, *E. nana*, and *E. sosorum* eyes followed the same protocol as above. Negative controls can be found in File S3 for opsin and File S4 for rhodopsin.

## Results

### Adult ocular histology and measurements from early stage and adult eyes

**Figure 1:**
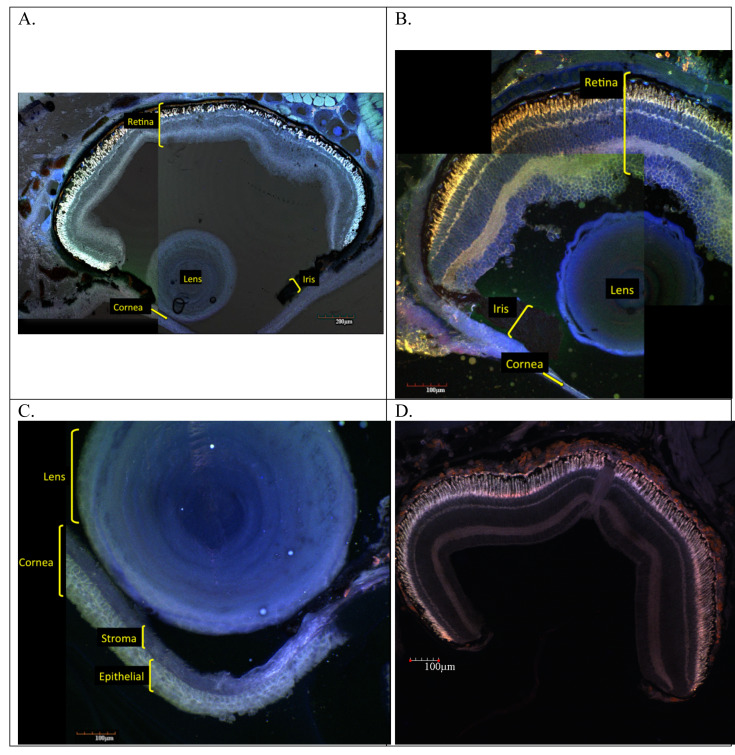
Sections of adult *E. nana* (A, C and D) and *E. sosorum* (B) eye. Using a laser scanning confocal microscope to detect autofluorescence from the sections, montages of images were produced using digitally applied pseudocolors to provide contrast. These images illustrate regions of the posterior eye showing well–developed retinal layers and pigment (A, B, D). The lens, cornea, and iris are also visible (A, B, C).

**Figure 2:**
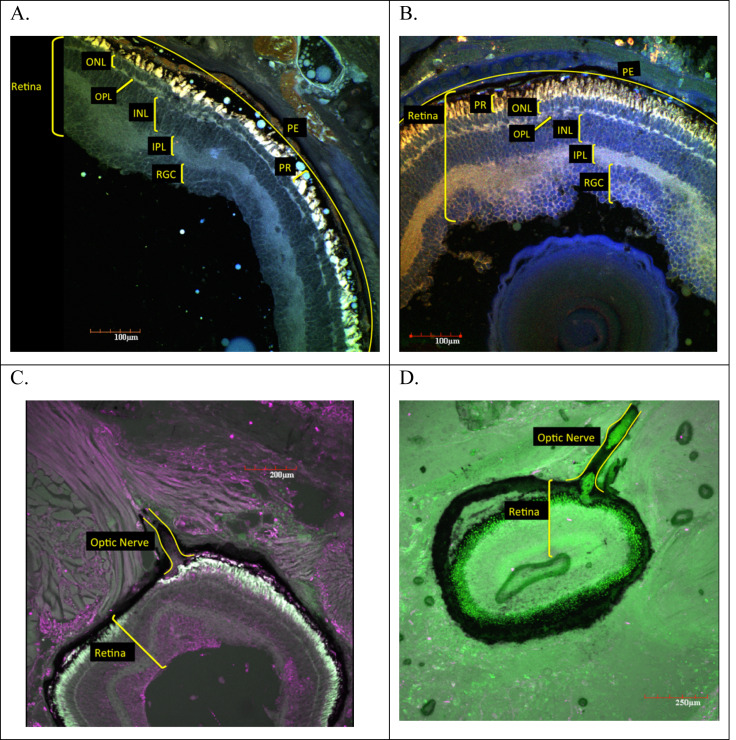
Ocular sections of adult *E. nana* (A, C) and *E. sosorum* (B, D). Using a laser scanning confocal microscope to detect autofluorescence from the sections, montages of images were produced using digitally applied pseudocolors to provide contrast. Ocular sections from adult *E. nana* (A and C) and adult *E. sosorum* (B and D) and associated retinal layers and optic nerve (C and D). Layers include pigment epithelium (PE), photoreceptor layer (PR), outer nuclear layer (ONL), outer plexiform layer (OPL), inner nuclear layer (INL), inner plexiform layer (IPL), and retinal ganglion cell layer (RGCL).

**Figure 3:**
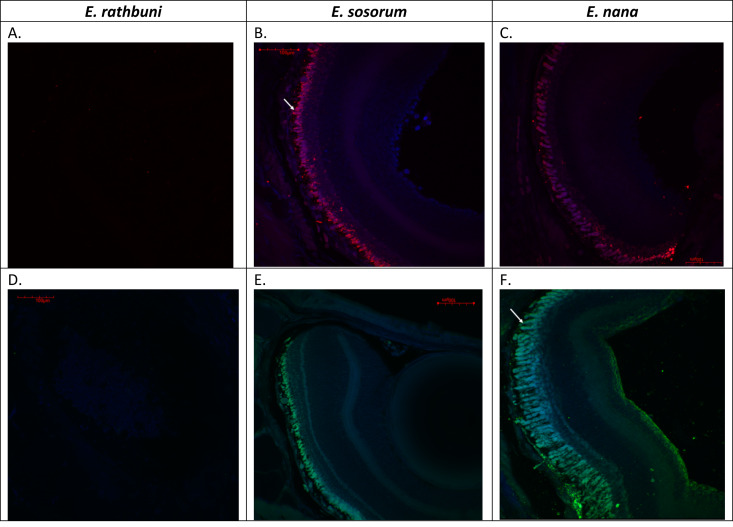
Adult *E. rathbuni*, *E. sosorum,* and *E. nana* retinal sections showing opsin and rhodopsin labeling. Images were acquired using a 20X water–immersion objective. Both surface species, *E. sosorum* (B & E) and *E. nana* (C & F) show labeling of opsin (A–C) and rhodopsin (D–F) in the photoreceptor layer of the retina (white arrows). No labeling is observed in the subterranean species *E. rathbuni* (A&D).

Examination of adult ocular sections taken from two surface species and a subterranean species revealed markedly different histology between the two ecotypes. Histological sections from the surface species *Eurycea nana* and *E. sosorum* revealed well-defined retinal layers, corneal layers, iris, and lens (Fig. 1). A closer examination of the retinas (Fig. 2) consistently revealed seven layers we identified as retinal ganglion cell layer (RGL), inner plexiform layer (IPL), inner nuclear layer (INL), outer plexiform layer (OPL), outer nuclear layer (ONL), photoreceptors (PR), and retinal pigment epithelium (RPE). Although a nerve fiber layer was only sometimes apparent (Fig. 1D), a well-defined optic nerve was observed in both species. In the surface salamanders, melanized tissue was found primarily in the RPE, the choroid, and the ciliary body of the iris; however, some dark pigmentation was also observed outside the sclera and surrounding the optic nerve (Fig. 2). We additionally observed opsin and rhodopsin labeling in the two surface species (*E. sosorum* and *E. nana*); this labeling was absent in the subterranean species *E. rathbuni* (Fig. 3). Labeling in the two surface species was localized to the photoreceptor layer of *E. sosorum* and E*. nana* (Fig. 3; white arrows).

**Figure 4:**
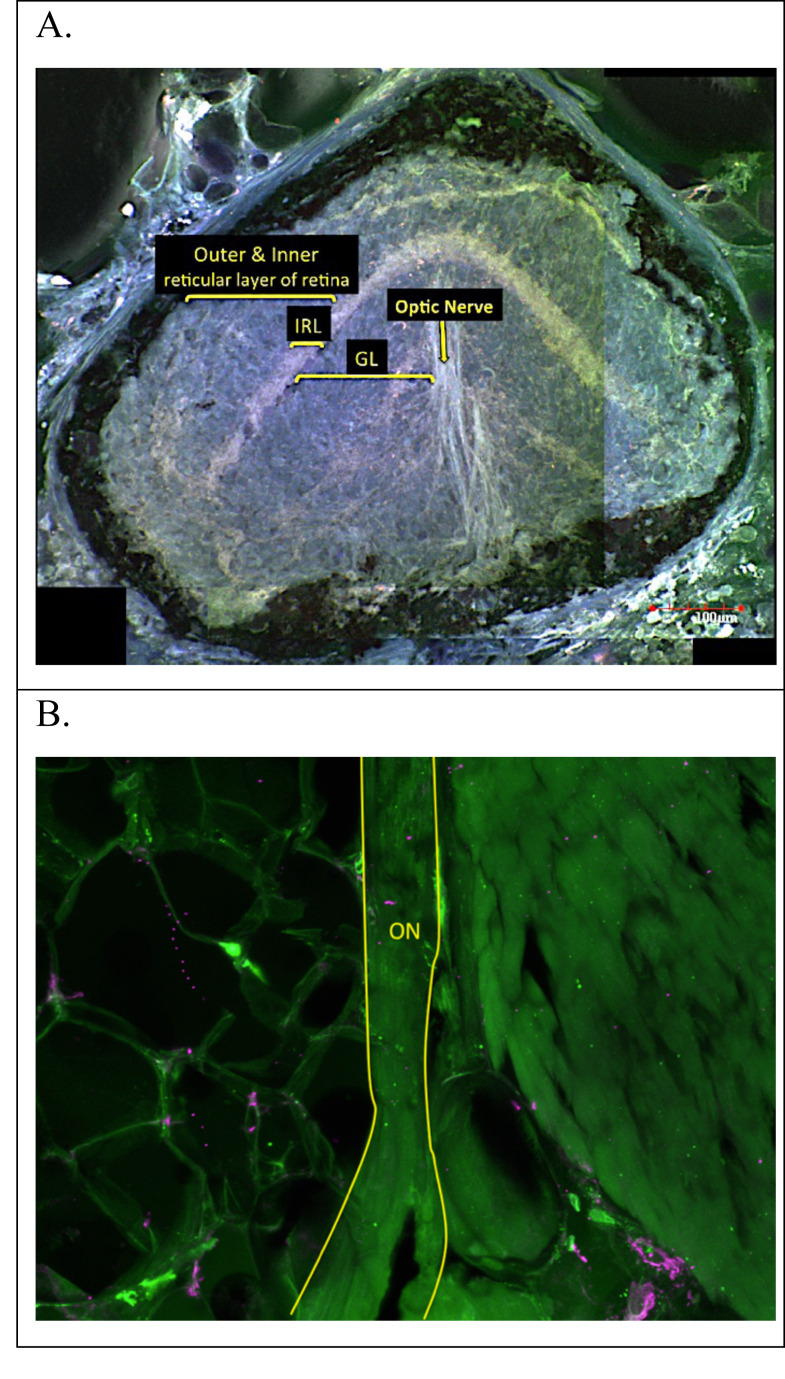
Adult *E. rathbuni* ocular sections. Sections showing undifferentiated tissue layers surrounded by pigment epithelium (A). The optic nerve is attached to the posterior region of the vestigial eye (A) and can also be seen at higher magnification and outlined in yellow (B). Sections are labeled after Eigenmann (1900) as follows: optic nerve (ON), ganglion layer (GL), inner reticular layer (IRL). outer and inner reticular layer of the retina.

Features previously described by Eigenmann (1900) for *Eurycea rathbuni* were identified and included (using his terminology) optic nerve (ON), three retinal layers, namely ganglion layer (GL), outer and inner reticular layer (O/IRL) and the pigment epithelium (PE). (Eigenmann may have used the term pigment epithelium (PE) because the pigmented layer in the eye of *E. rathbuni* appears to encompass the entire vestigial retina and is not restricted to its ancestral position sclerad to the photoreceptors as is the case for RPE.) No lens was identified in any adult *E. rathbuni*. A well-defined optic nerve was observed emanating from the eyes of *E. rathbuni* (Fig. 4). The entire ocular structure was surrounded by melanized tissue.

**Figure 5:**
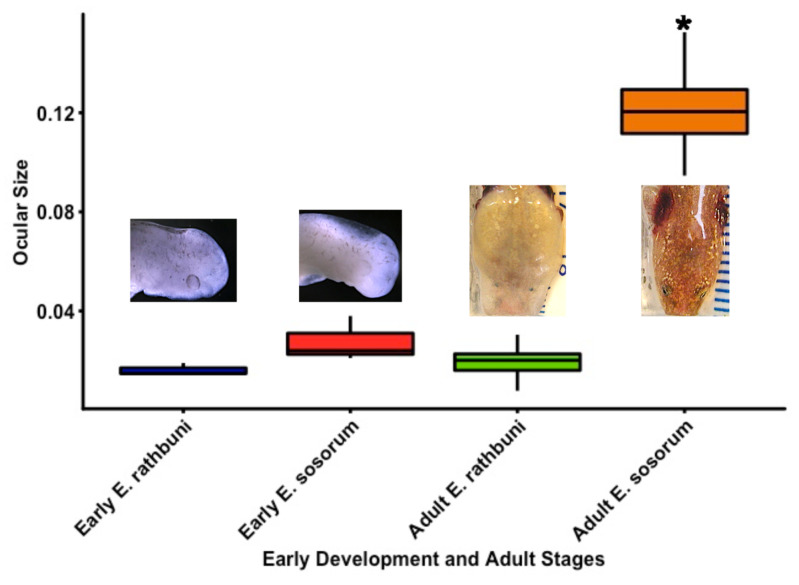
Normalized eye sizes for two species of salamander at different stages of development. Two developmental stages (early vs. adult) were measured and scaled to head size for two species from the central Texas *Eurycea* clade exemplifying subterranean (*E. rathbuni*) and surface (*E. sosorum*) optics. ANOVA and a post-hoc Tukey’s test revealed that the ocular size of the adult *E. sosorum* was statistically significantly larger than the early stage *E. sosorum* and early and adult *E. rathbuni*. In contrast, there was no statistically significant difference between the ocular length of adult *E. rathbuni* and either embryonic salamander.

There were no significant differences (*P* > 0.05) in the overall thickness of the retina or the thickness of component layers when comparisons were made between sections taken from *E. sosorum* and *E. nana* (File S5). The thickest layer of the retina in both species is the inner nuclear layer (INL), which contains the cellular nuclei of bipolar cells, horizontal cells and amacrine cells, and represents 22.9% of the retinal thickness in *E. nana* and 26.0% in *E. sosorum* (File S5).

We tested differences in eye size between *E. rathbuni* relative to *E. sosorum.* Measurements of the whole eye scaled to head length were obtained from animals early in development (stages 37 and 40) for *Eurycea rathbuni* (*n* = 3) and *E. sosorum* (*n* = 3) and from adult *E. rathbuni* (*n* = 34) and *E. sosorum* (*n* = 34) (Fig. 5). *E. nana* individuals were not included in this analysis as we did not have early developmental stages for this *species*. A one-way ANOVA and a post-hoc Tukey’s HSD test revealed a difference between adult *E. sosorum* and all other groups (Tukey’s HSD *P* < 0.001). There were no differences in the relative size of the eye between adult *E. rathbuni* and either species in their early developmental stages (Table 1).

### Developmental series and Pax6 localization

**Figure 6:**
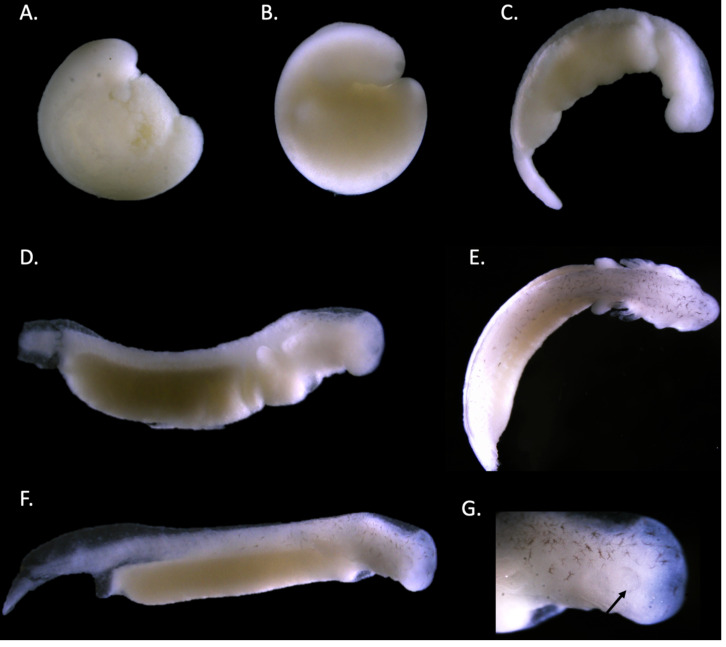
Staging of the Barton Springs salamander (*E. sosorum*). Stages are assigned based on morphology (Duellman & Trueb, 1986). Stages are as follows: Stage 21 (A). Stage 25–26 (B). Stage 31 (C). Stage 34 (D). Stage 37–38 (E). Stage 40 (F & G). Pigment accumulation (black arrow) around the developing eye in stage 40 is noted (G).

**Figure 7:**
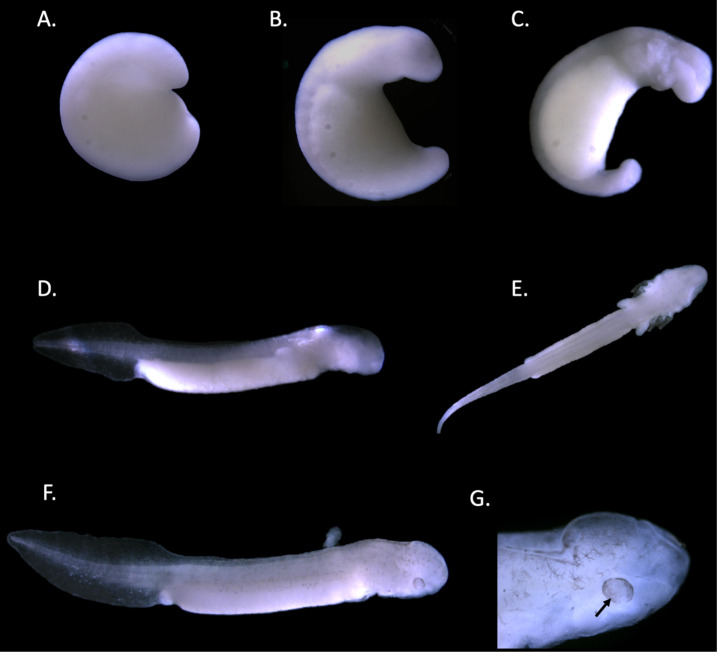
Staging of the Texas blind salamander (*E. rathbuni*). Stages are assigned based on morphology (Duellman & Trueb, 1986). Stages are as follows: Stage 21 (A). Stage 25–26 (B). Stage 31 (C). Stage 34 (D). Stage 37–38 (E). Stage 40 (F &G). Pigment accumulation (black arrow) around the developing eye in stage 40 is noted (G).

Staging was accomplished by following morphology for *E. sosorum* (Fig. 6) and *E. rathbuni* (Fig. 7). We were able to identify the following stages: stage 21 (embryos were defined as having neural folds closed to form neural tube), stages 25–26 (defined as having a prominent head, ear spot dorsal to the hyomandibular groove, and 9–10 somites), stage 31 (distinct gill folds), stage 37–38 (prominent gill folds, forelimb buds, and pigment migration from neural tube), and stage 40 (a prominent forelimb and hind limb bud, elongated and laterally compressed tail, pigmentation, and prominent eye spot) (Duellman & Trueb, 1986). The migration of melanocytes and concentrated pigmentation of the eye suggest ocular development (Figs. 6G, and 7G, black arrows).

**Figure 8:**
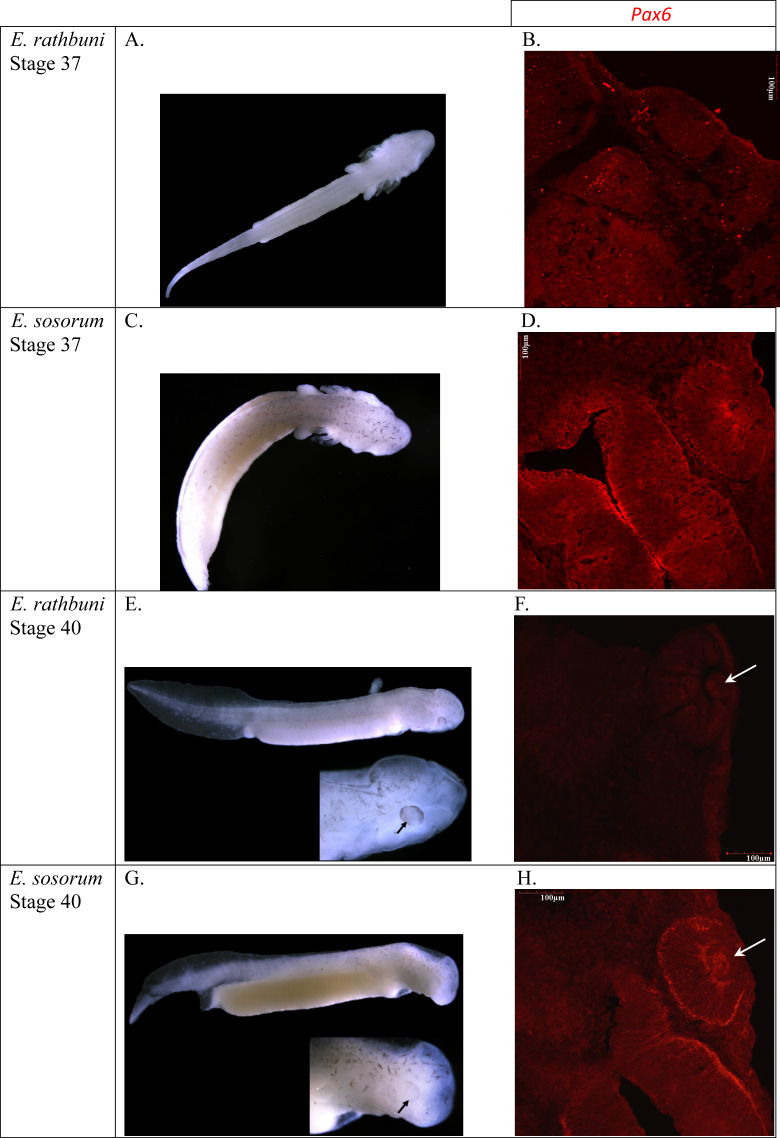
*E. rathbuni* and *E. sosorum* embryos at two stages of development and sections illustrating Pax6 labeling. The top two rows (A–D) of images show embryos at stage 37 (A & C) with their respective histological sections (B & D); the bottom two rows (E–H) of images show embryos at stage 40 (E & G) with black arrows indicating eye development. Histological sections for stage 40 (F & H) show labeling for Pax6 and lens development (white arrows). The image of a histological section through the anterior part of a stage 37 *E. rathbuni* embryo shows tissue positive for Pax6 labeling, where aggregates (not labeling; B) of secondary antibody are also evident (negative controls: File S2).

Pax6 protein is observed in the two phenotypes represented by *E. rathbuni* (subterranean phenotype) and *E. sosorum* (surface phenotype). Labeling of Pax6 is also observed in and around the midbrain, optic cup, and lens vesicle of both species. The labeling of Pax6 in stage 40 of *E. rathbuni* is noticeably reduced compared to stage 37 in the same species and to both developmental stages of *E. sosorum*. Pax6 is strongly expressed in the tissue surrounding the developing optic cup of *E. sosorum*, and labeling is particularly noticeable within the lens of *E. sosorum* at stage 40 (Fig. 8).

## Discussion

This study provides a foundation of descriptive ocular histology comparing three closely related species and two ecotypes, surface and subterranean. *Eurycea rathbuni* has drastically reduced eyes, a characteristic widely accepted as reflecting adaptation to subterranean life and exemplified by other stygobitic organisms, including other cave-dwelling salamanders (e.g., *Proteus anguinus*), cave-dwelling fish (e.g., *Astyanax mexicanus*), as well as extremely phylogenetically divergent invertebrates (Romero, 2009). *E. rathbuni* exhibits a few vestigial retinal layers surrounded by melanized tissue. Were light available to this stygobitic salamander, it likely would be unable to pass through the pigmented cells surrounding the eye to be detected by photoreceptors. Nevertheless, the optic nerve is still present in *E. rathbuni* (Fig. 3), suggesting a possible sensory function, but probably not vision.

Upon close examination of *E. rathbuni* histology, the feature identified by Eigenmann (1900) as an optic nerve penetrating to the center of the eye resembles the hyaloid canal. The hyaloid canal provides vascularization to the developing lens during early embryogenesis (Dunlop, Moore & Beazley, 1997). Early hyaloid vascularization occurs when the hyaloid artery and vein follow the optic fissure via the optic stalk distally, eventually reaching the optic cup and lens vesicle, where they provide the necessary vascularization for the continued development of the lens. We found that ocular development in *E. rathbuni* progresses to the point of an optic cup and lens vesicle (Fig. 8). Therefore, it is likely that hyaloid vascularization is present during development. Nevertheless, a structure resembling the optic nerve clearly exits the eye, raising the question of what its function could be, given the light-free environment these animals occupy in nature and the melanized tissue completely surrounding the reduced eye. The optic nerve could be a vestige of the developmental pathway and processes that also facilitate forebrain development.

We propose further investigations to track development and characterize these structures. When focusing on other regions of the eye like the retina, we could not distinguish retinal layers in the last stage of the subterranean species’ developing eye; however, we do observe an optic cup in both stages.

**Table 1:** One way ANOVA comparing eye size including early development and adult stages of *E. rathbuni* and *E. sosorum*.

	Early *E. rathbuni*	Early *E. sosorum*	Adult *E. rathbuni*
Early *E. sosorum*	*P* > 0.05	–	–
Adult *E. rathbuni*	*P* > 0.05	*P* > 0.05	–
Adult *E. sosorum*	^∗∗^*P* < 0.001	^∗∗^*P* < 0.001	^∗∗^*P* < 0.001

The black *Proteus anguinus parkelj* develops a lens and a photoreceptor layer of the retina. The blind *P. anguinus anguinus* develops a cluster of lens precursor cells and has partially developed photoreceptor disks (Kos, Bulog & Röhlich, 2001). A close relative to the Texas *Eurycea* clade, the Georgia blind salamander (*Eurycea wallacei*) retains a rudimentary lens in at least half of the individuals sampled by Brandon (1968). A number of stygobitic teleost systems have been explored, including *A. mexicanus* (Jeffery, 2009), *P. andruzzii* (Stemmer et al., 2015), and *Sinocyclocheilus anophthalmus* (Meng et al., 2013). Some terrestrial species like mammals (e.g., the genus *Spalax*), caecilians, and snakes show a range of underdevelopment in their eyes; the underdevelopment is thought to be associated with the dim-light environment of fossorial living (Carmona, Jiménez & Collinson, 2008; Simões et al., 2015). For the most part these species retain a lens, retinal layers, and some amount of photoreceptor development. These observations illustrate that early ocular development of the vertebrate eye is mostly conserved.

Phylogenetic studies suggest the ancestor of *Eurycea rathbuni* had well-developed eyes, similar to extant epigean species from this genus (e.g., *E. sosorum*) (Chippindale et al., 2000; Wiens, Chippindale & Hillis, 2003; Sweet, 1984). The surface species *E. nana* and *E*. *sosorum* have well developed retinal layers, including photoreceptors and retinal pigment epithelium, exhibiting ocular anatomy expected of species endowed with vision (Linke, Roth & Rottluff, 1876; Heatwole, 1998). These surface species exhibit a lens, cornea, iris, and a well-developed optic nerve. Taken together, it appears that all the ocular structures necessary to support vision are in place.

When eye size was compared among early developmental stages and adults of *E. rathbuni* and *E. sosorum*, no differences were observed between adult *E. rathbuni* and either species’ embryos (Fig. 4). This result suggests that the reduced eye size in *E. rathbuni* may reflect underdevelopment, i.e., a failure of development to progress. Fundamental knowledge of ocular anatomy has important implications for current research involving the central Texas *Eurycea*. For example, the full extent of visual function in the surface species may affect mate choice and predator or prey recognition (Fite, 1976; Roth, 1987). Future quantification of photoreceptors and their associated wavelength optima could elucidate the extent of color perception (Himstedt, Helas & Sommer, 1981; Korenyak & Govardovskii, 2013; Mohun & Davies Wayne, 2019) and the preferred active time during the day (e.g., nocturnal, diurnal, or crepuscular). Opsin and rhodopsin labeling in adult eye sections of all *E. nana* and *E. sosorum* was associated with the outer segment of the photoreceptors. The morphology of the outer segments suggested rods dominate the photoreceptor layer in both surface species. Rods function during scotopic, or low light, vision, while cones are mostly responsible for photopic (bright light and color) vision. The retention of rods and their photo-responsive pigment (rhodopsin) in amphibians adapted to dark habitats (e.g., caecilians and *Proteus anguinus*) may provide some maintenance of photoperiodic perception (Mohun & Davies Wayne, 2019; Kos, Bulog & Röhlich, 2001). However, we observe neither morphologically distinct photoreceptors nor visual pigment labeling in the subterranean *E. rathbuni*.

One objective of this study at the outset was to examine expression of the homeobox gene *pax6* during the development of the eye in surface and subterranean salamanders. Pax6 is well known to drive eye development in a range of widely divergent animals, including both vertebrates and invertebrates (Kumar & Moses, 2001). Unfortunately, we only had 1–2 sections for each species at each stage that were suitable for labeling and comparison—too few to quantify labeling. However, given the rarity of such specimens and the similarity in pattern to what has been observed in *Asytanax*, we offer a preliminary, descriptive comparison of Pax6 localization between *E. rathbuni* and *E. sosorum*.

Compared spatially, the localization of Pax6 proteins through development of *E. rathbuni* and *E. sosorum* is similar and follows what is expected during vertebrate neurulation. Specifically, *pax6* is expressed in the developing central nervous system, including the brain and eye (Wawersik & Maas, 2000). The continued expression of *pax6* and *vax1* genes is important as they encode transcription factors that bind with the enhancer sequence of the *α-crystallin* gene, which in turn encodes δ-crystallin proteins found in the lens (Wawersik & Maas, 2000). With noteworthy exceptions, if *pax6* expression is down regulated during the development of the lens, the lens will cease to develop (Carmona et al., 2010). In the subterranean fish *Asytanax mexicanus*, the down regulation of *pax6* gene consequent to upregulation of *shh* expression contributes to apoptosis of the lens, which stunts further retinal differentiation and results in the formation of vestigial remnants of retina (Jeffery, 2005).

The ocular histology of adult *Eurycea sosorum* reveals a well-organized retina, suggesting a functional eye and by extension canonical expression of morphogens associated with eye development. In the newt *Cynops pyrrhogaster*, *pax6* gene expression persists through adulthood and plays an important role in regeneration when the animal is subjected to retinal injury (Del Rio-Tsonis, CH & Tsonis, 1995). In *E. rathbuni* the presence of Pax6 protein is noted early in development at stage 37 and is spatially distributed in the developing brain and eye in a pattern similar to that seen in *E. sosorum*. However, Pax6 was not detected at stage 40, suggesting diminished expression in *E. rathbuni*. Otherwise, patterns of ocular development observed in *E. sosorum* also occur in *E. rathbuni*, particularly in the development of a lens in the subterranean *E. rathbuni*. Therefore, it appears that some degree of lens development may occur in all vertebrate systems that live in dim to no light environments, and also exhibit reduced optics. Lens development in dim to no light systems occurs in fossorial mammals, e.g., *Talpa occidentalis* (Carmona, Jiménez & Collinson, 2008); the cave and aquifer teleosts *Astyanax mexicanus* (Jeffery, 2009), *Phreatichthys andruzzii* (Stemmer et al., 2015), and *Sinocyclocheilus anophthalmus* (Meng et al., 2013); and cave and deep aquifer Caudates, *Proteus anguinus* (Durand, 1976), *E. wallacei* (Brandon, 1968), and now *E. rathbuni* as demonstrated here. This observation also brings to light the far-reaching constraint and convergence in the process leading to an underdeveloped eye. Together, both the development of a lens and the localization patterns of Pax6 observed in *E. rathbuni* suggest parallel ocular development with *A. mexicanus*.

Low levels of Pax6 may explain the underdevelopment of the eye of *Eurycea rathbuni*. Investigation of Pax6 and Shh protein levels in later stages of *E. sosorum* and *E. rathbuni* is needed to understand the completion of retinal development in *E. sosorum* and lens degeneration in *E. rathbuni*. Moreover, examining later stages would illuminate the molecular underpinnings of lens degeneration and enable one to specifically address the role of apoptosis as a means to eye regression as seen in *Astyanax mexicanus*. Importantly, the overall ontogeny and localization of the Pax6 protein during ocular development of the two salamander phenotypes parallel the two phenotypes explored in *A. mexicanus* (Jeffery, 2009). This parallel suggests that the salamanders examined in this study and the teleost fish examined by Jeffery (2009) may share a degree of convergent evolution in development and in the molecular mechanisms (*pax6*) responsible for the divergent ocular phenotypes in two vertebrate lineages, fish and salamander, occupying similar subterranean habitats. Studies incorporating intermediate stages are needed to determine the divergence of tissue and gene expression between the epigean and subterranean phenotypes, and if, as reported by Jeffery (2009), these early expression patterns lead to apoptosis of the lens.

## Conclusions

The comparative examination of ocular histology suggests *Eurycea nana* and *E. sosorum* are capable of vision while development of the retina in *E. rathbuni* is aborted prior to hatching, and the lens is lost at some point during ontogeny. We observed similar early ocular development between the two phenotypes, including the development of a lens in *E. rathbuni*. Taken together, parallels during early embryonic development were observed between the two phenotypes, whilst ocular morphology and histology in adults is drastically different. These results raise interesting questions about the evolution of subterranean phenotypes and the selective pressures they experience, how eyes are lost and what the molecular mechanisms responsible for ocular development and loss of eyes are.

As a vertebrate eye develops multiple genes are transiently expressed. Some of the most conserved are observed during early development (e.g., *pax6* and *shh*), and as tissues continue to differentiate, genes specific to those tissues are expressed (e.g., *cry* and *opsin*; (Zuber et al., 2003). In the evolution of a reduced eye, these gene networks are also thought to reflect molecular evolutionary processes. Studies have identified different networks associated with species exhibiting reduced eyes (Emerling & Springer, 2014; Simões et al., 2015; Protas et al., 2006). We are working to identify the molecular networks involved in eye development in the central Texas *Eurycea*.

This study provides a platform using a stygobitic tetrapod to understand the evolutionary developmental biology of eye reduction. Moreover, a non-transgenic tetrapod model may provide novel insight to the genes and their regulation in developing a healthy eye. In the future, we hope to use multiple species from this clade and sequencing approaches incorporating intermediate stages to better understand the evolution and underlying genetic mechanisms responsible for the diverse subterranean phenotypes.

## Supplemental Information

10.7717/peerj.11840/supp-1 Supplemental Information 1 Measurements of adult and early eye development for *E. rathbuni* and *E. sosorum* Each measurement is standardized by head length (E_H), for each species (sp) are adult (rath =E. rathbuni; sos =E.sosorum), and early development (early_rath = early developmental stage for *E. rathbuni*; early_sos = early developmental stage for *E. sosorum*).

10.7717/peerj.11840/supp-2 Supplemental Information 2 Negative controls of a stage 40 for *E. rathbuni* embryo (panels A and B) and *E. sosorum* (C and D) (A and C) show Hoechst (nuclear) staining. (B and D) show negative (no primary antibody) controls for Pax6 labeling. Images were taken with the same settings as experimental sections shown in Fig. 8 in the manuscript.

10.7717/peerj.11840/supp-3 Supplemental Information 3 Negative controls for opsin in adult *E. nana* (Panels A&B), *E. sosorum* (Panels C&D), and *E. rathbuni* (Panels E&F) (A, C, and E) show Hoechst (nuclear) staining. (B, D, and F) show negative (no primary antibody) controls for opsin labeling. Images were taken with the same settings as experimental sections shown in Fig. 3 in the manuscript.

10.7717/peerj.11840/supp-4 Supplemental Information 4 Negative controls for rhodopsin in adult *E. nana* (Panels A&B), *E. sosorum* (Panels C&D), and *E. rathbuni* (Panels E&F) (A, C, and E) show Hoechst (nuclear) staining. (B, D, and F) show negative (no primary antibody) controls for rhodopsin labeling. Images were taken with the same settings as experimental sections shown in Fig. 3 in the manuscript.

10.7717/peerj.11840/supp-5 Supplemental Information 5 Thickness of the retina and its component layers Retinal layers and their corresponding thickness in *E. nana*, and *E. sosorum*.

## Additional Information and Declarations

### Data Availability

The following information was supplied regarding data availability: The raw eye to head ratio measurements, control data Pax 6 images, control data for opsin images, control data for rhodopsin images and retinal thickness are available in the Supplemental Files.
