# Supplementary material for: Comparative development and ocular histology between epigean and subterranean salamanders (Eurycea) from central Texas
Source: PeerJ. 2021 Jul 28;9:e11840. doi: 10.7717/peerj.11840 (PMC8325428; doi:10.7717/peerj.11840)
Supplement: Supplemental Information 3 — (A, C, and E) show Hoechst (nuclear) staining. (B, D, and F) show negative (no primary antibody) controls for opsin labeling. Images were taken with the same settings as experimental sections shown in Fig. 3 in the manuscript. [file peerj-09-11840-s003.pdf]

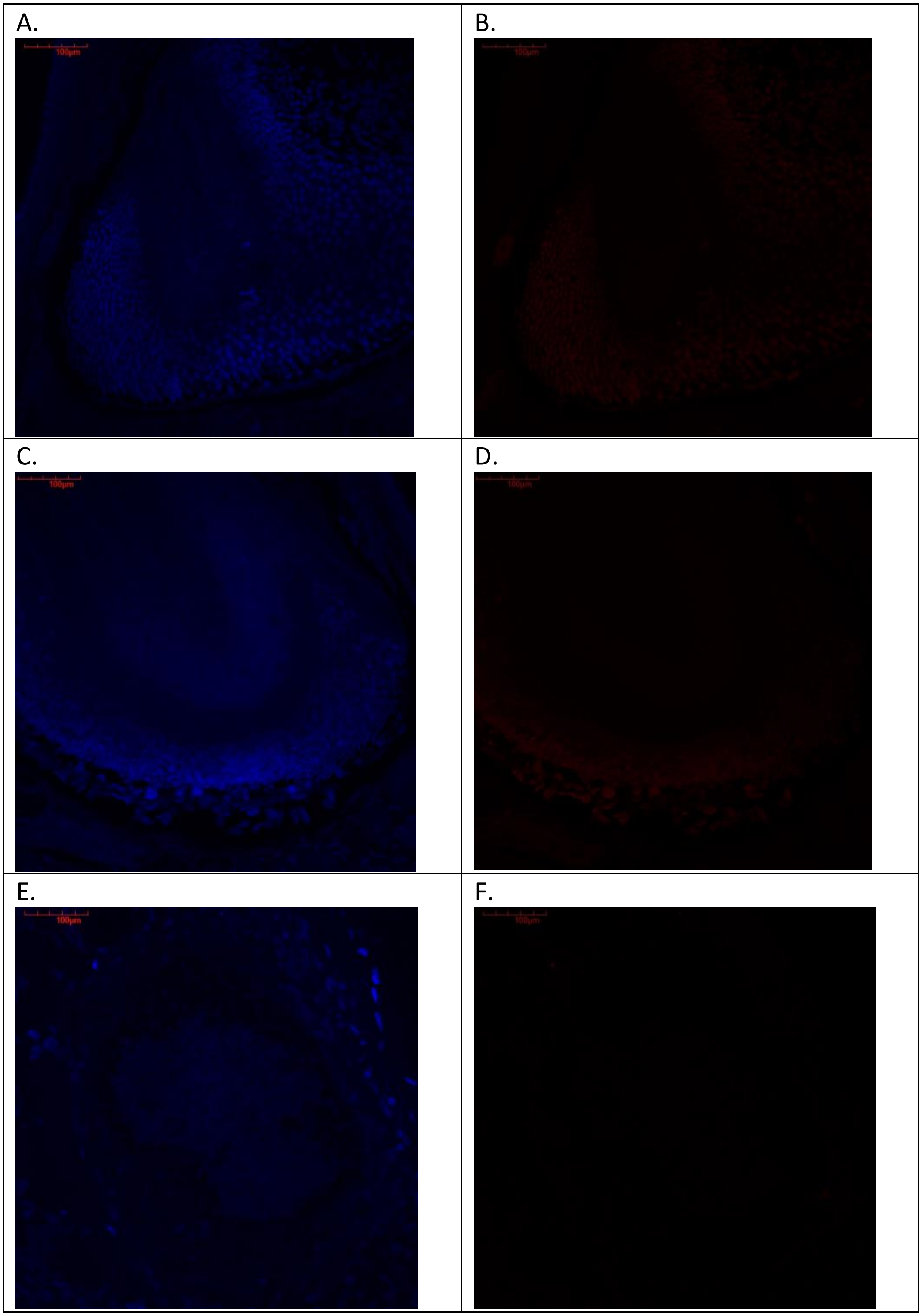

Figure ##. Negative controls for opsin in adult *E. nana* (Panels A&B), *E. sosorum* (Panels C&D), and *E. rathbuni* (Panels E&F). Panels A, C, and E show Hoechst (nuclear) staining. Panels B, D, and F show negative (no primary antibody) controls for opsin labeling. Images were taken with the same settings as experimental sections shown in Figure## in the manuscript.
