# Supplementary material for: Comparative development and ocular histology between epigean and subterranean salamanders (Eurycea) from central Texas
Source: PeerJ. 2021 Jul 28;9:e11840. doi: 10.7717/peerj.11840 (PMC8325428; doi:10.7717/peerj.11840)
Supplement: Supplemental Information 5 — Retinal layers and their corresponding thickness in E. nana, and E. sosorum. [file peerj-09-11840-s005.docx]

| **Table 1: Thickness of the retina and its component layers.** | | | |
| --- | --- | --- | --- |
|  | Mean^2^ Thickness ± SEM  (µm) | |  |
|  | *E. sosorum* | *E. nana* | P-value^3^ |
| RGCL | 50 ± 10 | 52 ± 3 | 0.9 |
| IPL | 34 ± 6 | 38 ± 5 | 0.7 |
| INL | 66 ± 5 | 69 ± 6 | 0.8 |
| OPL | 9.9 ± 0.9 | 9 ± 1 | 0.6 |
| ONL | 32 ± 3 | 29 ± 5 | 0.6 |
| RPEPRL | 49 ± 5 | 64 ± 4 | 0.08 |
| RET | 244 ± 7 | 260 ± 22 | 0.61 |
| ^1^RGCL = retinal ganglion cell layer; IPL = inner plexiform layer; INL = inner nuclear layer; OPL = outer plexiform layer; ONL = outer nuclear layer; RPEPRL = combined retinal pigment epithelium and photoreceptor layers; RET = entire retina  ^2^N = 3 individuals for all data.  ^3^P-values were computed from a two-tailed, Student’s T-test. | | | |
